# Effect of Selected Factors on the Serum 25(OH)D Concentration in Women Treated for Breast Cancer

**DOI:** 10.3390/nu13020564

**Published:** 2021-02-09

**Authors:** Agnieszka Radom, Andrzej Wędrychowicz, Stanisław Pieczarkowski, Szymon Skoczeń, Przemysław Tomasik

**Affiliations:** 1 Medical Laboratory Diagmed, Lwowska 20, 33-300 Nowy Sącz, Poland; a_radom@interia.pl; 2 Department of Pediatrics, Gastroenterology and Nutrition, Faculty of Medicine, Jagiellonian University Medical College, Wielicka 265, 30-663 Kraków, Poland; andrzej.wedrychowicz@uj.edu.pl (A.W.); stpiecz@wp.pl (S.P.); 3 Department of Oncology and Hematology, Pediatric Institute, Faculty of Medicine, Jagiellonian University Medical College, Wielicka 265, 30-663 Kraków, Poland; szymon.skoczen@uj.edu.pl; 4 Department of Clinical Biochemistry, Pediatric Institute, Faculty of Medicine, Jagiellonian University Medical College, Wielicka 265, 30-663 Kraków, Poland

**Keywords:** vitamin D deficiency, vitamin D measurement, vitamin D supplementation, breast cancer

## Abstract

Maintaining an optimal vitamin D concentration reduces the risk of recurrence and extends survival time in patients after breast cancer treatment. Data on vitamin D deficiency among Polish women after breast cancer therapy are limited. Thus, the aim of the study was the analysis of vitamin D status in post-mastectomy patients, considering such factors as seasons, social habits, vitamin D supplementation and its measurements. The study involved 94 women after breast cancer treatment. Serum vitamin D concentration was measured, and a questionnaire, gathering demographic and clinical data regarding cancer, diet, exposure to sun radiation, and knowledge of recommendations on vitamin D supplementation, was delivered twice, in both winter and in summer. The control group consisted of 94 age-matched women with no oncological history. In women after breast cancer treatment, 25-hydroxyvitamin D (25(OH)D) deficiency was much more frequent than in the general population. Only about half of the patients supplemented vitamin D at the beginning of the study. After the first test and the issuing of recommendations on vitamin D supplementation, the percentage of vitamin D supplemented patients increased by about 30% in study groups. The average dose of supplement also increased. None of the women that were not supplementing vitamin D and were tested again in winter had optimal 25(OH)D concentration. It was concluded that vitamin deficiency is common in women treated for breast cancer. Medical advising about vitamin D supplementation and monitoring of 25(OH)D concentration should be improved.

## 1. Introduction

Breast cancer is one of the biggest problems in public health. Globally, more than one million cases are diagnosed annually [1]. In terms of cancer mortality in women, breast cancer ranks in second position. In 2015, in the Polish population, breast cancer was the cause of 14.1% of deaths due to cancer; in 2016 it was already 14.5%, while in 2017 this percentage was 14.8% [2,3,4]. In the world, these proportions differ, varying significantly depending on race, latitude, and socioeconomic status [5].

The main biological function of vitamin D is the maintenance of homeostasis of calcium-phosphate management and the regulation of bone metabolism. It has been proven to play a role in the proper functioning of the immune, cardiovascular, and reproductive systems. In addition, vitamin D deficiency is associated with an increased incidence of type 1 and 2 diabetes, obesity, asthma, inflammatory bowel disease and cancer [6,7]. In 1990, Garland et al. were the first to demonstrate a negative relationship between total, average annual exposure to solar radiation and age-dependent mortality of breast cancer patients [8]. Many studies and meta-analyses have shown a relationship between vitamin D status and breast cancer risk [9,10,11,12]. However, in some of them, the negative correlation of breast cancer risk with 25-hydroxyvitamin D (25(OH)D) concentration was only found in retrospective studies and or in specific subpopulations of women with regard to menopausal status and ethnicity [13,14,15]. The discovery of the nuclear vitamin D receptor (VDR) and the demonstration of its presence in cancer cells gave rise to research into the role of vitamin D in the development and course of cancer [16,17]. Vitamin D has anticancer properties by affecting inflammation, cell’s growth, maturation, and proliferation. It inhibits angiogenesis and metastasis ability, reduces the number of estrogen receptors, inhibits the expression of adhesion molecules, regulates miRNA expression, modulates the hedgehog signaling pathway, and induces breast cancer cell apoptosis in vitro and in vivo [18,19,20]. In vitamin D deficiency, there is a dysregulation of cells’ growth and proliferation, and facilitation of neoangiogenesis and carcinogenesis. At least 35 genes are regulated with vitamin D in breast tissue, and their activity is associated with invasiveness and cancer metastasis [21].

In recent years, great attention has been paid to maintaining proper 25(OH)D concentration in a healthy population as well as especially in people after cancer treatment. Despite numerous reports on the relationship between serum 25(OH)D concentration and the risk of breast cancer, its progression and distant prognosis, there are no uniform guidelines on what doses of vitamin D and what serum concentrations should be considered appropriate in both healthy and oncological patients. Guidelines of American Cancer Society/American Society of Clinical Oncology recommended calcium (1200 mg/d) and vitamin D (600–1000 IU/d) supplementation in breast cancer patients from 50 years of age to reduce bone loss-related mortality [22]. In turn, the guidelines of the European Society of Medical Oncology (ESMO) state that the daily supply of vitamin D in breast cancer patients should be in the amount of 1000–2000 IU [23]. Polish guidelines for supplementation and treatment of vitamin D for healthy people and risk groups of deficiencies recommend that adults should take 800–2000 IU vitamin D per day depending on body weight. In the risk groups of vitamin D deficiency to which cancer patients belong, supplementation should be carried out under the control of laboratory determinations 25(OH)D to maintain an optimal concentration between 30 and 50 ng/mL [24]. According to Polish Standards of Nutritional Treatment in Oncology (2015), indications for supplementation of vitamin D include a documented deficiency in the blood or typical clinical characteristics of vitamin D deficiency [25]. There are also no global uniform laboratory criteria for determining vitamin D deficiency based on its blood concentrations. According to the recommendation of the US National Academy of Medicine, adults should supplement vitamin D to maintain serum concentrations of 25(OH)D above 20 ng/mL (50 nmol/L) [26,27]. The American Association of Clinical Endocrinology (AACE) and the Endocrine Society recommend serum concentrations of 25(OH)D ≥30 ng/mL (75 nmol/L) as sufficient [28]. The 2016 Guidelines of the German Food Society state that the desired serum vitamin D concentration is >20 ng/mL (>50 nmol/L) [29]. Although prevention and treatment of vitamin D deficiencies is recommended in the daily practice of doctors and clinical nutritionists, this problem is often overlooked in oncological patients due to the lack of uniform guidelines for such patients, and determining which specialist is responsible for implementing and monitoring vitamin D supplementation. Therefore, the aim of the study was to evaluate the serum 25(OH)D concentrations of women after being treated with breast cancer according to the seasons, eating and social habits, vitamin D supplementation, and the recommendations of the attending physicians and the effect of vitamin D determinations on improving vitamin D status in subsequent testing and changing behavior to obtain and maintain the recommended serum vitamin D concentration.

## 2. Materials and Methods

### 2.1. Studied Groups

The patients who underwent radical treatment of breast cancer (mastectomy) were included in the study. The groups were separated depending on the season in which the patients were included in the study. Group A—62 women, in whom the first questionnaire and vitamin D concentration determination was carried out in winter (December 2016–January 2017). In these patients, the research procedure was repeated in the summer (July–August 2017). Group B—32 women with the first survey and determination of vitamin D concentration made in the summer (July–August 2017) and repeated in the winter (December 2017–January 2018). Patients from groups A and B, after first tests, received laboratory interpretation of the 25(OH)D concentration result, and additionally, in the case of results beyond the reference values, patients were informed about the need to obtain doctor’s advice. Control group (93 women) was recruited among the participants of the regional screening vitamin D testing program for people at risk—at the age of 40 and above. The program was carried out in November–December 2017. Detailed data are available in Supplementary material File S1: Recruitment of patients.

### 2.2. Survey

The study used an indirect research method in the form of a questionnaire, which each of the respondents completed twice, in winter and summer, before or after (not longer than a month) the determination of vitamin 25(OH)D. The survey asked, inter alia, about basic demographic and anthropometric data (age, height, and body mass); clinical, cancer-focused (age at which breast cancer was diagnosed, presence of neoplasms including breast cancer among relatives, methods of breast cancer treatment, radiation therapy in the past), data on the reproductive system (age at the first menstruation, age at menopause, birth of the first child, use of hormone replacement therapy (HRT)); eating habits (frequency of consumption of vitamin D-rich products); exposure to natural solar/UV radiation (average daily exposure time, protection against UV radiation); knowledge of recommendations on vitamin D supplementation in oncological patients and recommendations provided by the attending physician on vitamin D supplementation, use of vitamin D supplementation—the dose taken all year round. Wallace’s rule of nines was used to estimate the percentage of the body area that patients expose to sunlight. Food products rich in vitamin D were selected based on the Nutrition Standards for the Polish population [30] and Polish recommendations for the prevention of vitamin D deficiency [31]. Additional data are available in Supplementary Materials File S2: Survey validation; File S3: Personal data anonymization.

### 2.3. Laboratory Determinations

Venous blood was collected from all subjects fasting, in the morning. Determinations of vitamin D concentration were performed using the Elecsys Vitamin D total II tests (Roche Diagnostics, Mannheim, Germany) on the Integra cobas e411 analyzer (Roche Diagnostics, Mannheim, Germany) according to the manufacturer manual.

### 2.4. Statistics

The distribution was verified by the D’Agostino–Pearson normality test. The results are presented using the descriptive statistics. To evaluate relationships between continuous variables with normal distributions, the parametric Pearson correlation test was used. In other cases, the non-parametric test was used—Spearman’s rank correlation. The analysis of variance (ANOVA) or Friedman’s rank test was used to compare the groups in terms of a measurable feature. To assess the differences between the selected factors in the group A and B, the student’s *t*-test was used for normally distributed variables, and the Mann–Whitney U-test (both for independent variables) for variables with a different distribution. In the case of comparing the dependent variables, the paired *t*-test was used and Wilcoxon signed-rank test for a non-parametric data. The chi square test was used to compare the distribution of data categorized in studied groups. The results in which *p* < 0.05 were considered statistically significant. Statistica 13 (TIBCO Software Inc., Palo Alto, CA, USA) and MedCalc 15.8 (MedCalc Software Ltd., Ostend Belgium) were used for analysis.

#### Sample Size Calculation

The sample size for laboratory determinations of vitamin D was calculated from data obtained from the winter and summer tests of first studied group A (women treated from breast cancer), using “Sample size for before-after study (Paired *t*-test)” calculator from the University of California San Francisco, Clinical & Translational Science Institute website. The calculated effect size was 10.7, the standard deviation of the change in the outcome was 15.1 with alpha (type I error rate) set at 5% and a beta (type II error rate) of 10%. Calculation using the T statistic and non-centrality parameter showed a sample size of 23, and approximation using the Z statistic instead of the T statistic showed required a sample size of 21 paired measurements.

The recommended sample size for the survey is 214 and above. With our studied group (size *n* = 92; combined group A and B), the margin of error was 8.17% instead of the recommended 5%, with a confidence level of 90%.

## 3. Results

### 3.1. Demographical and Anthropometrical Characteristic of Studied Groups

Study groups A and B as well as the control group were similar in terms of age, body mass and BMI. Detailed data are presented in Table S1, in Supplementary Materials. Also important clinical data are available in Supplementary Materials Table S2: Characteristics of patients related to the reproductive system and Table S3: Characteristics of patients related to the cancer disease.

### 3.2. Vitamin D Concentration in Studied Patients

**Figure 1:**
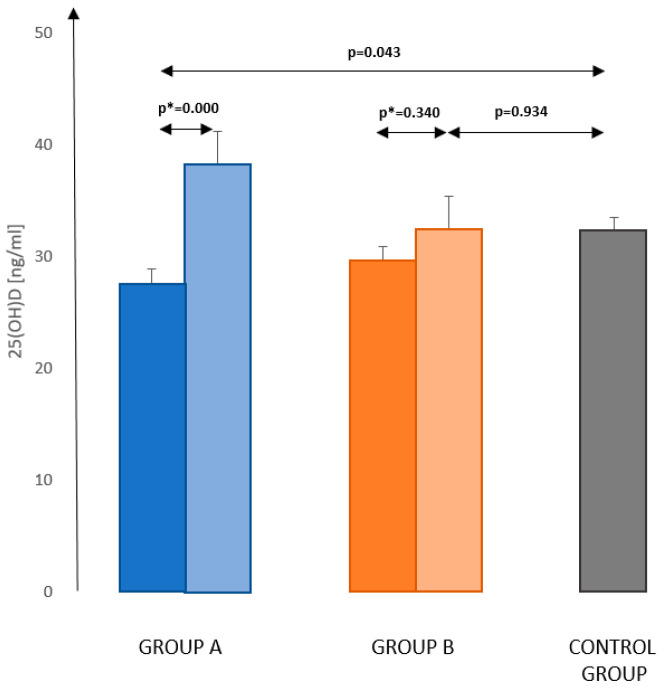
Mean concentrations of 25(OH)D (±SD—error bars) in the healthy control group and the groups of women treated for breast cancer. Values before inclusion into study—dark (left) bars in the pair and during second testing half year later—pale bars (right) in the pair. Group A—women treated for breast cancer tested the first time in winter; Group B—women treated for breast cancer tested the first time in summer. Comparison of the results in the control group (obtained in winter) with the results of studied groups in winter. p*—paired *t*-test; *p*—*t*-test.

The mean concentration of vitamin D in group A from the first sampling was 27.6 ± 14.1 ng/mL, and six months later, the mean concentration was 38.3 ± 12.2 ng/mL (*p* = 0.000 paired *t*-test). In group B, in the first sampling, the concentration of vitamin D was 29.6 ± 13.6 ng/mL, and after six months, it increased to 32.4 ± 13.3 ng/mL (*p* = 0.340 paired *t*-test). In the control group, the mean vitamin D concentration (determined in winter) was 32.2 ± 14.4 ng/mL (Figure 1).

**Figure 2:**
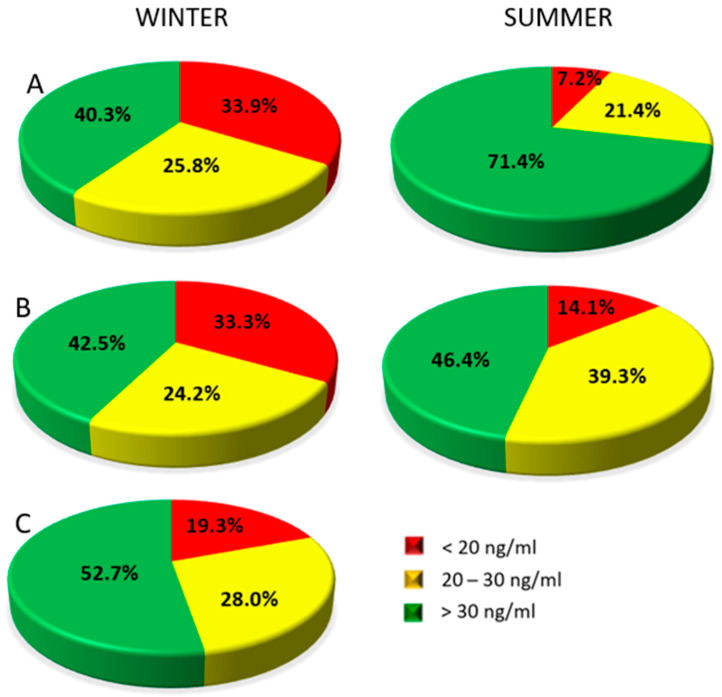
Percentage of women with vitamin 25(OH)D concentrations below the reference value range, with suboptimal and optimal values in Group A—women treated for breast cancer tested the first time in winter; testing during winter—left disc, testing during summer—right disc. (**A**), Group B—women treated for breast cancer tested the first time in summer; testing during winter—left disc, testing during summer—right disc (**B**), and the control group (**C**) (testing during winter only).

In group A, in the results obtained in winter, the serum concentration of 25(OH)D in 33.9% of patients was below 20 ng/mL (below the reference range), in 25.8% it was within 20–30 ng/mL (suboptimal level), while in 40.3% of patients it was above 30 ng/mL (normal value). In the results obtained in the summer (second sampling), serum 25(OH)D concentration below 20 ng/mL was observed in 7.1% of patients, in 21.4% it was in the range of 20–30 ng/mL, and in 71.4% of patients, it was above 30 ng/mL (Figure 2A).

In group B, in the results obtained from the first sampling (summer), serum concentration of 25(OH)D in 33.3% of patients was below 20 ng/mL, in 24.2% it was within 20–30 ng/mL range, while in 42.5% of patients it was above 30 ng/mL. In the results obtained from the second sampling (winter), serum 25(OH)D concentration below 20 ng/mL was found in 14.3% of patients, in 39.3% it was within the range of 20–30 ng/mL, and in 46,4% of patients were above 30 ng/mL (Figure 2B).

In the control group (sampling in winter), the serum concentration of 25(OH)D in 19.4% of patients was below 20 ng/mL, in 28.0% it was within the range of 20–30 ng/mL, while 52.7% were above 30 ng/mL (Figure 2C).

### 3.3. The Concentration of Vitamin D and Diet

In the group of patients examined in winter, significantly lower concentrations of vitamin D were noted in women who consumed milk 1–2 times a week compared to those who did not drink milk at all or occasionally (respectively, 23.1 ng/mL and 30.7 ng/mL, *p* = 0.0145). On the other hand, in the study group tested for the first time in the summer, patients who consumed eggs 1–2 times a week had higher vitamin D values compared to those who did not eat eggs or ate them sporadically (29.4 ng/mL and 17.4 ng/mL, respectively *p* = 0.0522). None of the other analyzed diet components had a statistically significant effect on the 25(OH)D concentration in the studied patients. The detailed information about consumption of food rich in vitamin D in studied groups is delivered in Supplementary material Table S4: The frequency of consumption of foods rich in vitamin D per week in the combined group A + B and control group, before entering the study and Table S5: Number of patients consumed food rich in vitamin D before first and second testing in combined A + B groups.

### 3.4. Supplementation of Vitamin D in Studied Patients

At the time of enrollment in the study, more than half of patients after breast cancer treatment did not supplement with vitamin D. In group A, it was 51.6% (*n* = 32), and, in group B, 56.2% (*n* = 18). According to the data from the questionnaires performed during the second sampling, after obtaining the vitamin D concentration result from the first testing, the percentage of patients supplemented vitamin D in group A increased to 75.8% (*n* = 47), also, in group B, the percentage increased to 75.0%. The average dose of supplemented vitamin D also increased. In group A, the average dose of vitamin D taken before the first test (in winter) was, on average, 1500 units per day (range from 200 to 4000 units). Before the next test, the average intake of vitamin D was slightly above 1700 units per day (range from 500 to 4000 units). In group B, the average intake of vitamin D before the first examination was nearly 2000 units per day (range from 1000 to 8000 units). Before the next sampling, it increased, on average, almost to 2500 units per day. Detailed data about the concentration of vitamin D depending on supplementation are presented in Table 1.

**Figure 3:**
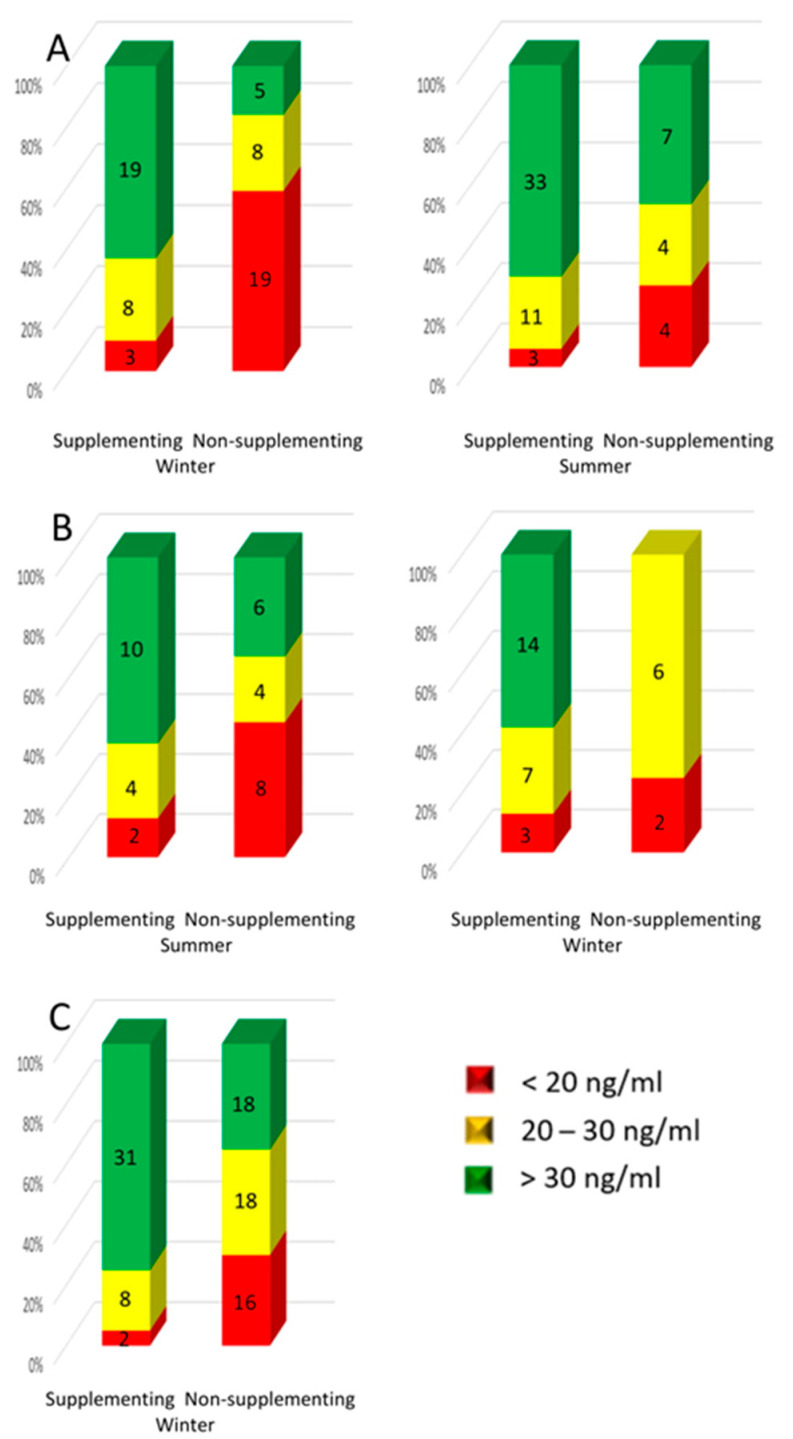
Percentage of patients in the healthy control group and the groups of women treated for breast cancer, with deficiency, suboptimal and optimal vitamin D levels depending on supplementation of vitamin D and season—winter/summer. Group A—women treated for breast cancer tested the first time in winter (**A**); Group B—women treated for breast cancer tested the first time in summer (**B**), and the control group tested during winter only (**C**). The number of persons in every subgroup is listed on the bars.

In group A, during the first part of the study (winter), the deficit of 25(OH)D was found in 10.0% (*n* = 3) of patients who took the pharmacopeial form of vitamin D and in 59.4% (*n* = 19) of patients who did not use supplementation. The suboptimal level was found in 26.7% (*n* = 8) of patients taking vitamin D preparations and in 25.0% (*n* = 8) of patients not taking pharmacopeial vitamin D. Recommended vitamin D concentration values (>30 ng/mL) were found in 19 patients (63.3%) taking pharmacopeial vitamin D and 5 people (15.6%) not taking any vitamin D supplementation. In the results obtained in the second part of the study (summer; group A), 25(OH)D concentrations below 20 ng/mL were found in 6.4% (*n* = 3) of patients who took the pharmacopeial form of vitamin D, and in 26.7% (*n* = 4) of non-supplementing patients; the suboptimal level was found in 23.4% (*n* = 11) of people taking vitamin D supplements and in 26.7% (*n* = 4) of patients not taking the pharmacopeial vitamin D. Optimal vitamin D concentration was found in 70.2% (*n* = 33) of people using pharmacopeial vitamin D and in 46.6% (*n* = 7) of people not using vitamin D supplementation (Figure 3A).

In group B, in the results of first sampling (summer), the vitamin D deficit was found in 12.5% (*n* = 2) patients who took the pharmacopeial form of vitamin D, and 44.5% (*n* = 8) in the group non-supplementing pharmacopeial vitamin D; the suboptimal level was found in 25.0% (*n* = 4) of people taking and 22.2% (*n* = 4) of those not taking vitamin D preparations. The optimal value of vitamin D concentration was found in 62.5% (*n* = 10) of people using pharmacopeial vitamin D and in 33.3% (*n* = 6) of people not supplementing vitamin D. In the results obtained in the second sampling (winter), the 25(OH)D deficiency was found in three patients (12.5%) who took the pharmacopeial form of vitamin D, and two non-supplementing (25.0%). The suboptimal level was found in seven (29.2%) patients taking vitamin D preparations and in six (75.0%) patients not taking the pharmacopeial vitamin D. The optimal vitamin D concentration was found in 14 patients (58.3%) taking the pharmacopeial vitamin D and in none of the patients who did not supplement vitamin D (Figure 3B).

In the control group, the vitamin D deficient were two (4.9%) persons who supplemented with vitamin D and 16 (30.8%) who did not supplement. The suboptimal level was found in eight (19.5%) people using vitamin D supplementation and in eighteen (34.6%) patients without such a support. The optimal values of vitamin D concentration were found in thirty-one (75.6%) people taking the pharmacopeial vitamin D and in eighteen (34.6%) people who did not supplement vitamin D (Figure 3C).

The percentages of women supplementing vitamin D in groups A and B at the first sampling with deficiency, suboptimal concentration, and optimal values, were similar (respectively, group A—10.0%, 26.7%, 63.3%; group B—12.5%, 25%, 62.6%). In the control group, among women supplementing vitamin D, the percentage of persons with the optimal vitamin D results was significantly higher (75.6%). On the other hand, among the non-supplementing patients in group B, there was a significantly lower percentage of results indicating vitamin D deficiency (44.5%) and a higher percentage indicating suboptimal and optimal (22.2 and 33.3%, respectively) than in group A (59.4%, 25.0%, and 15.6%, respectively).

### 3.5. Knowledge of Recommendations on Vitamin D Supplementation

**Figure 4:**
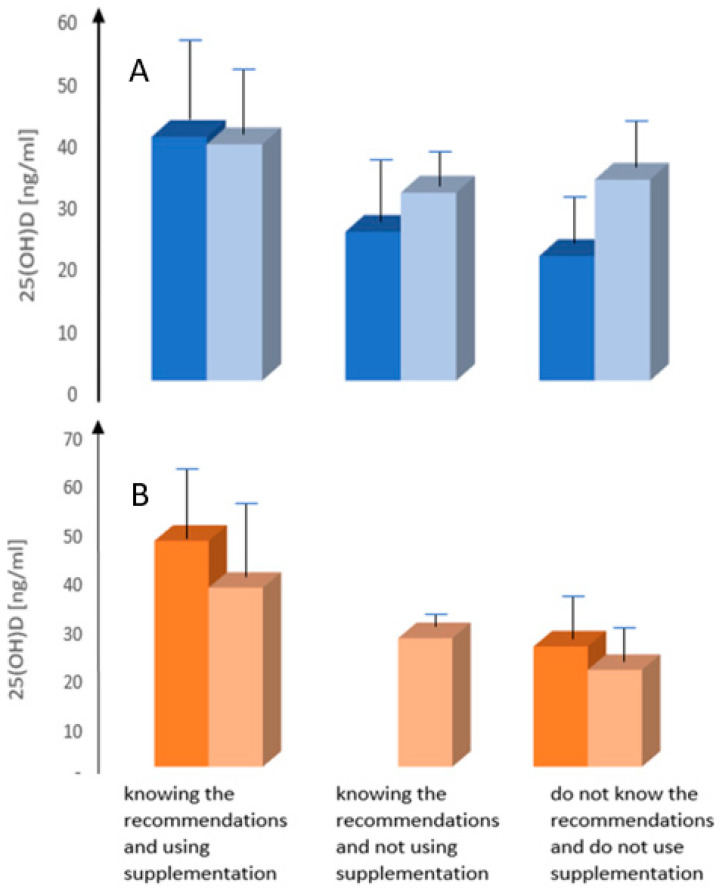
Mean serum 25(OH)D concentration (±SD—error bars) in the groups of women treated for breast cancer depending on the knowledge of vitamin D supplementation guidelines and supplementation of vitamin D. (**A**)—Group A—women treated for breast cancer tested the first time in winter. (**B**)—Group B—women treated for breast cancer tested the first time in summer. Dark (**left**) bars in the pair—values and data before inclusion into the study; pale bars (**right**) in the pair values and data during second testing half year later.

In group A, 14 (22.6%) patients declared that they knew the recommendations regarding vitamin D supplementation in women with breast cancer before entering the study. Among them, 10 (71.42%) declared the use of vitamin D supplementation. However, only five patients (35.7%) had a determined 25(OH)D concentration in the past. The average value of vitamin D concentration in group A, among those who knew the recommendations and used vitamin D supplementation, was 39.3 ng/mL, and, among women who knew the recommendation, but did not use vitamin D supplementation, the average concentration was 24.0 ng/mL, while the average concentration of vitamin D, in women who did not know the recommendations and did not use vitamin D supplements, was lower—20.1 ng/mL (Figure 4A).

In the second series of studies carried out in the summer, 39 (62.9%) patients declared knowledge of the recommendations regarding vitamin D supplementation in women with breast cancer, of which 92.3% (*n* = 36) declared the vitamin D supplementation. The average value of vitamin D concentration in the group of respondents knowing the recommendations and using vitamin D supplementation was 38.1 ng/mL, and among women who knew the recommendation, but did not use vitamin D supplements, this value was, on average, 30.3 ng/mL. Similarly, the average concentration of vitamin D in women who still did not know the recommendation and did not use vitamin D supplementation was 32.4 ng/mL (Figure 4A).

In group B, four patients (12.5%) declared knowledge of the recommendations regarding vitamin D supplementation in women with breast cancer before entering the study (summer). All the patients who knew the recommendations stated that they were using vitamin D supplements, and three of them (75%) had measured 25(OH)D concentration in the past. The mean vitamin D concentration among patients familiar with the recommendations and taking vitamin D supplements was 46.2 ng/mL. The mean concentration of vitamin D in women who did not know the recommendations and did not use vitamin D supplements was 24.6 ng/mL (Figure 4B).

In the next series of studies carried out in winter, 18 (56.3%) patients declared that they knew the recommendations regarding vitamin D supplementation in women with breast cancer, of which 15 (83.3%) used vitamin D supplements. The average value of vitamin D concentration in those who knew the recommendations and used vitamin D supplements was 36.6 ng/mL, and among women who knew the recommendation, but did not use vitamin D supplements, this value was, on average, 26.3 ng/mL. The mean concentration of vitamin D in women who did not know and did not use vitamin D supplements was 19.8 ng/mL (Figure 4B).

Only 15 women from group A and B had determined vitamin D which constitutes 15.9% of all examined patients. In addition, a similar percentage of women from the control group had previously undergone such a laboratory test (15.1%, *n* = 14).

**Figure 5:**
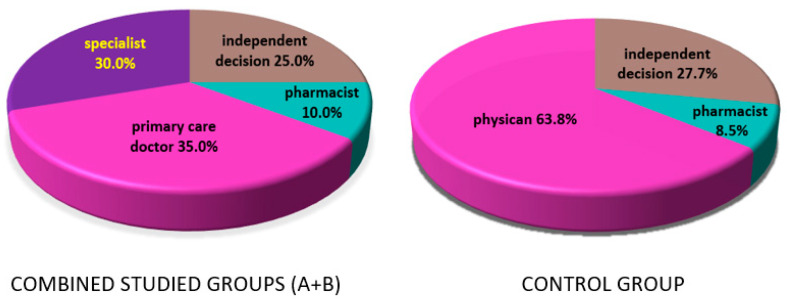
Sources of information on vitamin D supplementation in women treated for breast cancer before joining the program (combined group A in winter and group B in summer)—**left** disc; and in healthy control group—**right** disc. The detailed percentages are listed in the figure.

Vitamin D supplementation was recommended to patients in group A and B by GPs in 35.0% of cases (*n* = 14), specialists in 30.0% of cases (*n* = 12) (including oncologists, neurologists, endocrinologists, gynecologists, diabetologists, internists), and pharmacists in 10.0% of cases (*n* = 4). Some patients made their own decisions to start supplementation (25.0%; *n* = 10) without any medical professional advice. In the control group, vitamin D intake was most often recommended by a physician (63.8%; *n* = 30) or a pharmacist (8.5%; *n* = 4), and was also, in some cases, an independent decision of the patients (27.7%; *n* = 13) based on information obtained from media broadcasts and friends (Figure 5).

### 3.6. Exposure of Patients to Solar Radiation

In groups A and B, most of the patients declared that they protected themselves from the sun in summer (72.6% (*n* = 45) and 71.8% (*n* = 23), respectively); in the control group, the percentage of patients protecting themselves from the sun was lower—59.1% (*n* = 55); (*p* = 0.27, chi^2^ test). Most patients, after breast cancer treatment, protected themselves from the sun by wearing appropriate clothing (73.4%); 26.6% used only—or additionally—cosmetics with UV filters. In the control group, more than half of the patients used cosmetics with UV filters (50.7%) as basic or additional protection.

Patients from both studied groups (A + B), who did not protect themselves against solar radiation, according to their assessment, spent, on average, 151 ± 101 min in the sun daily during the summer, and patients from the control group spent 108 ± 101 min in the sun daily (*p* = 0.13).

There were no differences in vitamin D concentrations measured during the summer between the group of patients avoiding the sun (wearing appropriate clothing and/or using cosmetics with UV filters; 34.9 ± 13.9 ng/mL) and those who declared that they did not use any protection and even willingly sunbathed (34.1 ± 11.1 ng/mL).

### 3.7. Correlation of Vitamin D Concentration with Age

There was no statistically significant correlation between the age of the patients and the vitamin D concentration determined at the first measurement in the combined both studied groups (A + B) (R = 0.010, *p* = 0.91) and in the control group (R = 0.19, *p* = 0.068).

### 3.8. Correlation of Serum Vitamin D Concentration with Diet

To assess the effect of diet on 25(OH)D concentration, the data obtained from all patients during the winter period were analyzed to avoid bias due to endogenous production after exposure to sunlight. The correlation of vitamin D concentration in winter in all subjects with the total consumption of foods rich in vitamin D (fish, eggs, milk) was not statistically significant (R = 0.041; *p* = 0.585). Additionally, the correlation was assessed in the group of people who did not supplement vitamin D in order to ignore the effect of supplementation on the concentration of vitamin D. Again, in this case, no significant correlation was found (R = 0.004, *p* = 0.969).

### 3.9. Correlation of the Declared Tanning Time with the Concentration of Vitamin D

**Figure 6:**
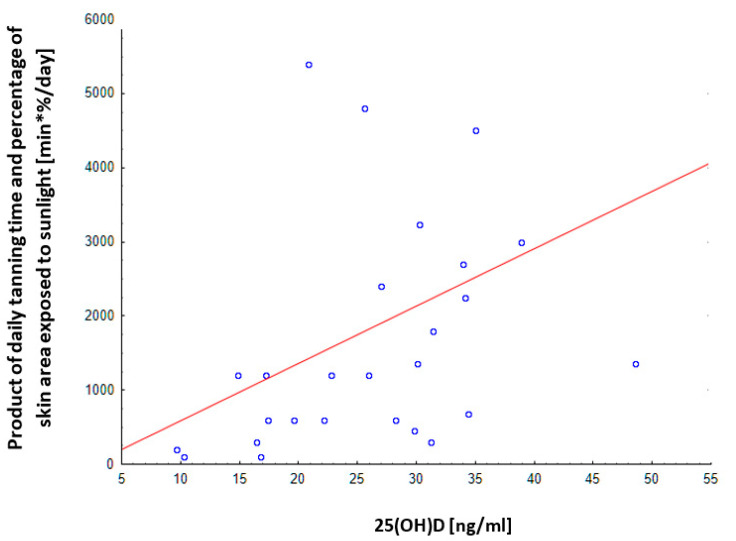
Correlation of the product of the declared tanning time and the percentage of the skin area exposed to sunlight with the concentration of vitamin D determined in the summer period in all surveyed people without vitamin D supplementation. *n* = 27, Pearson’s R = 0.584, *p* = 0.0014.

To analyze the influence of sunlight on vitamin D concentration, such a relationship was analyzed in patients who did not supplement vitamin D by correlating the time and area of skin exposed to the sun with the concentration of vitamin D determined in the summer months. The result was statistically significant—R = 0.584, *p* = 0.0014 (Figure 6).

## 4. Discussion

Recently, hypovitaminosis D has been recognized as one of the risk factors for breast cancer, and the concentration of vitamin D in the blood becoming a prognostic factor [21]. The results of meta-analyses show that higher serum 25(OH)D_3_ concentration determined after breast cancer diagnosis correlates with lower mortality due to breast cancer. In particular, patients with 25(OH)D_3_ in the highest quartile had approximately half the mortality rate compared to those with vitamin D in the lowest quartile [32]. There are also many reports on the relationship between the serum concentration of vitamin D and the achieved clinical results of breast cancer treatment and risk of recurrence [33,34]. The above-described relationship between the concentration of vitamin D and the risk of breast cancer or recurrence, as well as therapeutic success in the event of this disease, indicates the need to pay attention also to this factor. It can be consciously easily modulated by the time spent in the sun and diet, and it is also possible to administer this vitamin as pharmacopeial medication [32].

Vitamin D deficiency (serum 25(OH)D concentration < 20 ng/mL or <50 nmol/L) in the European population is a common phenomenon. The reports of the European Calcified Tissue Society estimate that 30–40% of the population of Central, Eastern, and Southeastern Europe is vitamin D deficient [35]. National studies showed even worse data [36,37]. Vitamin D deficiency is also common in the population of breast cancer patients, occurring in 23.0% to 95.6% of patients [38]. In the present study, a deficiency defined as 25(OH)D concentration below 20 ng/mL was demonstrated in the first sampling in 33.9% of patients in group A and 33.3% of patients in group B. Suboptimal concentration at the same time was found in 25.8% of patients in group A and 24.2% of patients in group B. In the control group, vitamin D deficiency was found in 19.3% patients, and suboptimal levels were observed in 28.0%. Andersen et al. showed that 30% of females in a group of American patients, after breast cancer treatment, were vitamin D deficient [39]. In addition, Apoe in a similar group showed that 62% of patients had 25(OH)D concentration below 30 ng/mL [40]. Mechado et al. tested 209 Brazilian women after breast cancer treatment and 26.2% had vitamin D deficiency and 55.6% had suboptimal levels [41]. These results confirm several published sets of data about the higher prevalence of vitamin D deficiency in the population of people with cancer. Furthermore, it should be remembered that serum 25(OH)D concentration above 20 ng/mL ensures the proper functioning of the calcium-phosphate metabolism and bone density, but only concentrations above 30 ng/mL ensure the optimal amount of vitamin D for other bodily functions, including proper functioning of immune mechanisms, as well as obtaining a protective effect in the context of neoplastic diseases [42].

Endogenous production stimulated by solar radiation is the primary source of vitamin D and is determined by geographical factors (latitude, the ozone layer, cloud cover, albedo), season, and individual factors (genetic, including skin phototype, time spent outdoors, body area exposed to the sunlight, applying cosmetics with UV filters, etc.) [43,44,45]. It is assumed that over 90% of the vitamin D requirement in summer is covered by the endogenous conversion of previtamin into provitamin D in the skin due to UVB radiation [46,47].

The present studies confirmed the seasonality of changes in 25(OH)D concentration in patients after breast cancer treatment. After excluding from the analysis the patients who declared supplementation with vitamin D, the tests performed for the first time in winter showed a higher percentage of patients with vitamin D deficiency compared to the tests performed in summer (58% and 44%, respectively). Additionally, in the group tested firstly in the summer, a significantly higher percentage of patients (33%) with optimal vitamin D concentrations was found than in patients tested first time in winter (16%). A similar seasonality in 25(OH)D concentration in patients after treatment for breast cancer has been demonstrated in other studies. Acevedo et al. found that the concentrations of 25(OH)D in women with breast cancer in summer were significantly higher compared to the tests performed in winter (*p* = 0.0322) [48]. Eliassen et al. also demonstrated the dependence of 25(OH)D concentration, in patients with breast cancer, on the time of year in which the measurement was performed [49]. A study of serum 25(OH)D concentration in 1940 women by Shi et al. during the first 6 months after breast cancer diagnosis showed higher 25(OH)D concentration in summer and fall [34].

Exposition to sunlight is an easily modifiable factor, but is season dependent. In the latitude of central Europe (between 30 and 55 degrees latitude), approximately half an hour of exposure to solar radiation in the mid-afternoon period during the summer, three times a week with bare limbs, is sufficient to obtain a serum concentration of 25(OH)D equal to or higher than 20 ng/mL (50 nmol/L) in 90% of the white population. The solar UVB radiation from October to March is weakened by an extended path through the atmosphere. As a result, in this period of the year, the skin synthesis of vitamin D is weaker and insufficient to cover human needs [45]. In addition, the change in lifestyle in recent decades, which has diminished exposure to sunlight, is of great importance in this respect [50]. According to the generally prevailing opinion, “excessive exposure” (for which there is no definition) to the sun is a factor causing skin cancer. That has led to a situation where most people believe that they should avoid sun radiation and over-shield the body or use cosmetics with UV filters [51]. The results of my research indicate that the majority of patients from groups A and B avoid sun radiation (72% (*n* = 45) and 73% (*n* = 23), respectively): two-thirds in the form of covered clothing, and the rest only, or additionally, used creams with protective filters. Despite such protection, there were no differences in vitamin D concentrations determined in summer between the group of patients declaring no use of protection from the sun and those who avoided the sun. That may result from a longer overall time spent outdoors by the latter women. The percentage of the body area protected with a UV filter, the SPF factor and the thickness of the cream layer applied to the skin should be also taken into account. At the same time, a statistically significant correlation was demonstrated (R = 0.584, *p* = 0.0014) between the time and area of the skin exposed to sunlight in patients who did not supplement vitamin D and the vitamin D concentration determined in the summer.

It is estimated that an average of 100–200 IU (2.5–5.0 μg) of vitamin D is consumed daily with the diet, which is 15–30% of the requirement [52,53]. Therefore analyzing data from the survey, it was not surprising that none of the women (*n* = 6) who decided not to take pharmacological supplementation, only to enrich their diet with natural products rich in vitamin D during a second sampling in a winter, did obtain an optimal vitamin D level. The results significantly confirm that it is impossible to supply an adequate amount of vitamin D in the latitude of Central Europe only through the diet in the winter months when there is no skin synthesis of vitamin D.

The lack of a statistically significant correlation between the diet and the concentration of vitamin D in the serum (R = 0.041; *p* = 0.585) may result from the widespread enrichment of various foods, which the study participants may not have pointed out or may not know.

Supplementation with pharmacopeial preparations is a relatively easy and inexpensive method of delivering vitamin D. The importance of vitamin D supplementation in long-term follow-up of breast cancer patients was shown in several papers. For instance, it was shown that the mortality due to breast cancer in patients who started supplementation after cancer diagnosis was 20% lower compared to the group not supplementing vitamin D, and the reduction in mortality was 49% in patients who started supplementing with such supplementation within no more than 6 months after diagnosis [54].

In the present study, during enrollment in the study, vitamin D supplementation was used by less than half of the patients (group A 48.4%; *n* = 30, group B 43.8%; *n* = 14). Patients who did not supplement vitamin D had significantly lower 25(OH)D concentration, regardless of the season. On the other hand, supplementation increased the percentage of women whose 25(OH)D concentration exceeded 20 ng/mL. Similar results were found in the group of 332 Swiss women after treatment for breast cancer. Only 133 patients took calcium supplementation with vitamin D or vitamin D alone. However, in many patients, despite the supplementation (800 IU), the vitamin D level remained suboptimal [55].

There are no uniform global guidelines on what vitamin D doses and serum concentrations should be considered as a target in the group of patients after breast cancer treatment. The National Comprehensive Cancer Network recommends that cancer patients should maintain 25(OH)D concentration above 30 ng/mL [56]. The American Cancer Society/American Society of Clinical Oncology recommends vitamin D supplementation in patients with breast cancer starting from the age of 50 at a dose of 600–1000 IU/day [22]; in turn, ESMO recommends higher daily doses of vitamin D in this group of patients, amounting to 1000–2000 IU/day [23]. It should be noted that both societies recommend the above doses to prevent bone loss related to the nature of the disease and the side effects of treatment, and not to prevent a recurrence. Since 2018, the Polish group of experts recommends vitamin D supplementation under the control of serum concentrations so that it should be between 30 and 50 ng/mL in groups with the risk of vitamin D deficiency, including, among others, the population of breast cancer patients. Supplementation should be carried out using daily doses of vitamin D between 800 and 2000 IU for adults, differentiated depending on its supply in the diet and current weight [24]. In addition, several scientists, based on the analysis of epidemiological studies of cancer risk, its progression, recurrence, and mortality, recommend higher doses, to maintain higher 25(OH)D concentration between 30-40 ng/mL, up to 60 ng/mL [56,57,58].

The percentage of patients declaring knowledge of the recommendations in the field of vitamin D supplementation in women with breast cancer before inclusion in this study was low—22.6% in group A and 12.5% in group B. During the second test, 62.9% and 56.3% of women in groups A and B declared knowledge of the recommendations regarding vitamin D supplementation. In addition, the percentage of people using vitamin D supplements increased in the group where knowledge of the recommendations was declared (during the first sampling in group A—71.42%; in the second—92.3%). Patients who did not know the recommendations and did not use supplementation had lower mean serum concentrations of 25(OH)D compared to those who knew the recommendations and supplemented, and even those who knew the recommendations but did not supplement vitamin D. Most of the surveyed women obtained information on the recommended principles of vitamin D supplementation from doctors; 35% from specialists and 30% from primary care physicians. Pharmacists advised supplementation in every tenth patient. Overall, 25% of patients made decisions about supplementation independently. More than half of the respondents (56.6%) had the dosage determined by a doctor, every fourth patient was advised by a pharmacist, and the remaining 23.3% of patients had chosen a vitamin D dose themselves.

Similar data were obtained in other countries. In Ireland, in 2005 only 15.5% of patients with breast cancer were instructed by a doctor about the need for supplementation and received a prescription for vitamin D. Six years later, this percentage increased to 36.9% of patients and the average daily dose of prescribed vitamin D was 857 IU/day [59]. In the USA, at the beginning of the 21st century, only 56% of breast cancer patients after chemotherapy-induced menopause received information on the recommended supplementation with vitamin D and calcium [60]. The best results in communicating the need for vitamin D supplementation in women after breast cancer treatment were reported in Croatia. According to a prospective 3.5-year study by Bosković et al., 75.7% of patients with breast cancer received a prescription for vitamin D and calcium from a medical oncologist. Unfortunately, a large group of patients, after breast cancer treatment (40–80% depending on the center where the women were treated), do not follow medical recommendations on vitamin D supplementation [61].

The presented data show that, in Poland and many other countries, a lot of patients diagnosed with breast cancer do not obtain information on the supplementation of vitamin D from attending doctors (oncologists, endocrinologists, gynecologists and GPs). Kimiafar et al. unequivocally showed that patients with breast cancer expect information about the disease, its course, prognosis, rehabilitation process, and variants of diagnostic and therapeutic procedures. However, they would most like to understand the possible side effects therapy, and allowed diets. Despite the growing awareness of physicians about the needs of patients with cancer, many patients still feel that they receive insufficient information or information that is unclear or incomprehensible. That is why patients often use alternative sources of information, such as the internet, books, and other patients, to obtain information on how to use supplements or compose the right diet [62].

All these data indicate the need for education of both patients and doctors about the benefits of maintaining optimal 25(OH)D concentration, especially in the group of patients treated for breast cancer. Whether vitamin D supplementation in oncological patients is the domain of oncology specialists or primary care physicians should be clearly defined.

Some studies show that patients, after breast cancer treatment, more often use vitamin D supplements when they are tested for the 25(OH)D concentration. In the large prospective The Sister Study, at the first testing, the regular use of vitamin D supplement was declared by 56% of women. Along with testing, participating women were informed about the planned vitamin D determination after a few years. During the second determination of vitamin D, regular supplementation was declared by 84% of women, and mean 25(OH)D concentrations were higher, due to the greater number of women who reported using vitamin D supplements [63].

The present study also showed an increased number of women treated for breast cancer supplemented vitamin D, after testing the 25(OH)D. The patients received laboratory interpretation of the result and were advised to obtain detailed recommendations, including recommended doses of supplementation and laboratory monitoring of vitamin D concentrations, from a doctor. When enrolled in the study, more than half of the patients from groups A and B did not supplement vitamin D, while, during the second testing, the percentage of people supplementing vitamin D increased to over 75% in both study groups.

According to this study and reports of other authors, vitamin D supplementation must be adapted to the season, lifestyle, and individual characteristics of a person, including the individual ability to absorb vitamin D [35]. Even advanced algorithms that allow individualized dosages of vitamin D, e.g., depending on age, body weight, or menopausal status, do not always bring the expected effects [64]. It is difficult to determine the optimal dosage due to the supply of vitamin D in the diet and endogenous production after exposure to solar radiation, the use of cosmetics with filters, the amount of fat tissue, skin pigmentation, and air pollution [65]. Therefore, in the Polish Standards of Nutritional Treatment in Oncology (2015), vitamin D supplementation should be ordered based on the result of laboratory determination of this component in the blood [25]. Similar recommendations were issued in 2018 by the Spanish Society of Oncological Medicine (SEOM) [66].

The data of the present study show that only about 15% of women from the study and control groups had their 25(OH)D concentration determined before being included in the research program. Similar results were obtained by Andersen et al. in a population of 553 American women diagnosed with breast cancer no later than 2 years before study enrollment. Interestingly, it was assessed whether conventional medicine physicians and practitioners of alternative medicine monitor the concentration of 25(OH)D in the group of patients treated conventionally, but supplemented with alternative medicine therapies. It was found that women who used complementary therapies in addition to conventional medicine had more frequently measured blood vitamin D compared to patients using only conventional medicine (30% vs. 16%) [39]. It can be assumed that doctors of natural medicine attach more importance to supplementation and holistic compensation than doctors ordering targeted pharmacological treatment.

The first measurement of 25(OH)D concentration during this study, and discussing the result with the patients and indicating the necessity to contact the attending physician in the case of deficiencies, prompted the patients to compensate for the deficits. There was a 26.73% reduction in the number of vitamin D deficient women in the next testing in group A and by 19.04% in group B, regardless of the season of the first and second tests. It was associated with an increase in the percentage of patients who used vitamin D supplementation by half and with higher mean concentration of 25(OH)D in the second measurement compared to the first.

Although supplementation with vitamin D under the control of laboratory determinations is the best solution, it should be remembered that laboratory tests are inconvenient for the patient and increase the cost of treatment. However, the price of vitamin D determinations is almost traceable to the costs of oncological treatment in the case of recurrence, and social costs of the patient’s death.

The limitation of the study may result from the use of the questionnaire in research proceedings. Patients participating in the study may have given the wrong answer due to difficulties in understanding the questions, or may have knowingly concealed the truth. Bearing in mind the above issues, a pilot was conducted along with the validation of the survey, the aim of which was to eliminate the factors affecting the credibility of the survey by using control questions and adjusting the questions contained in the survey so that they were as understandable as possible for the respondents (data available in supplements). A lack of control group analysis in summer slightly increases the uncertainty of some comparisons; however, the most important are relationships inside the same study group (tested twice). The influence of tumor location and type as well as the applied additional treatment during and after mastectomy was not analyzed and may be a source of potential bias. The number of patients in the study is sufficient for comparisons of laboratory data inside groups. The relatively low number of survey participants could be a source of uncertainty of data from the questionnaire.

## 5. Conclusions

- Women with treated breast cancer are more likely to develop vitamin D deficiency than the healthy population.
- The seasons, and the time and area of the skin exposed to solar radiation, influence the concentration of 25(OH)D in women after treatment for breast cancer.
- Diet is not important in maintaining the proper concentration of vitamin D in women after treatment for breast cancer.
- Supplementation with vitamin D significantly improves its status in women after treatment for breast cancer; therefore, oncologists and GPs should recommend it to patients.
- Knowledge of recommendations on vitamin D supplementation among patients after treatment for breast cancer is directly related to the more frequent use of supplementation and less frequent 25(OH)D deficiency.
- Laboratory monitoring of vitamin D concentration has a positive effect on maintaining optimal vitamin D concentration in women after breast cancer treatment.
- Routine vitamin D testing should be introduced to the screening panel in follow-up patients after breast cancer treatment.

## Figures and Tables

**Table 1:** Average 25(OH)D concentration in women treated for breast cancer and in healthy persons in relation to vitamin D supplementation and season.

	*n* Supplementing/ *n* Non-Supplementing	Patients Supplementing Vitamin D (ng/mL)	Patients Non-SupplementingVitamin D (ng/mL)	*p*
Group A winter	30/32	34.6 ± 14.2	22.4 ± 11.9	*p* = 0.0006
Group A summer	47/15	38.9 ± 12.2	34.8 ± 12.1	*p* = 0.24
Group B summer	16/18	36.8 ± 15.4	24.0 ± 9.0	*p* = 0.012
Group B winter	24/8	35.3 ± 13.7	22.8 ± 5.3	*p* = 0.001
Control group winter	41/36	38.3 ± 16.3	27.6 ± 10.9	*p* = 0.0006

Notes: Group A—women treated for breast cancer tested first time in winter; Group B—women treated for breast cancer tested the first time in summer; *n*—sample size; the variables are presented as mean ± SD.

## Data Availability

The data presented in this study are available on request from the corresponding author. The data are not publicly available due to General Data Protection Regulation (GDPR) and lack of such a permission from participants.

## Supplementary Materials

The following are available online at https://www.mdpi.com/2072-6643/13/2/564/s1, File S1: Recruitment of patients; File S2: Survey validation; File S3: Personal data anonymization; Table S1: Anthropometric characteristics of the patients; Table S2: Characteristics of patients related to the reproductive system; Table S3: Characteristics of patients related to the cancer disease; Table S4: The frequency of consumption of foods rich in vitamin D per week in the combined group A + B and control group, before entering the study; Table S5: Number of patients consumed food rich in vitamin D before first and second testing in combined A + B groups.
