# Supplementary material for: Effect of Selected Factors on the Serum 25(OH)D Concentration in Women Treated for Breast Cancer
Source: Nutrients. 2021 Feb 9;13(2):564. doi: 10.3390/nu13020564 (PMC7915136; doi:10.3390/nu13020564)
Supplement: Supplementary file 1 [file nutrients-13-00564-s001.zip › nutrients-1060971-supplementary materials/File S1 Recruitment of patients.docx]

Initially, 112 women after oncological treatment of breast cancer were qualified for the study groups.

BMI> 40 (n=1)

n=112

Age < 30 lat (n=1)

n=111

n=110

Lack of consent (n=4)

Do not followed up examination (n=11)

n=106

n=95

Death (n=1)

n= 94

Figure. Inclusion to studied groups

Initially, 141 women at the age above 50 were qualified for the control group.

Lack of consent (n=12)

n=141

age < 50 lat (n=19)

n=129

n=110

BMI<18,5 (n=7)

n=103

Cancer in medical history (n=10)

n=93

Figure. Inclusion of patients to control group.
