# Supplementary material for: Effect of Selected Factors on the Serum 25(OH)D Concentration in Women Treated for Breast Cancer
Source: Nutrients. 2021 Feb 9;13(2):564. doi: 10.3390/nu13020564 (PMC7915136; doi:10.3390/nu13020564)
Supplement: Supplementary file 1 [file nutrients-13-00564-s001.zip › nutrients-1060971-supplementary materials/File S2 Survey validation.docx]

Survey validation

Twenty women were asked twice, two weeks apart, to complete the same questionnaire. The repeatability of the questionnaire was checked by determining the Spearman's rank correlation coefficient between the results obtained for the same people in the first and second interviews, which was part of the validation of the questionnaire. The following criteria were used (according to Hoelscher, Am Diet Association, 2003):

0.70 -0.80 acceptable level

0.81-0.90 sufficient level

> 0.91 very good level

The results of the Spearman's R correlation coefficient for individual questions between the pairs of surveys ranging from 0.861 to 0.965. Some of the questions to which the patients had problems with giving an o answer were corrected.

Control questions were also used to verify the answers given by the respondents

1. Familial history of cancer question, control question - first degree relative's breast cancer. Inconsistent responses were considered to show a first-degree relative who was diagnosed with breast cancer, while at the same time in the question about the neoplasm occurring in the family, the answer about no cases was obtained.

2. The question about the use of vitamin D supplementation, as a control question, the number of months in which vitamin D is supplemented was asked. The number of months in which vitamin D supplementation is used was considered inconsistent, when in the question about the use of vitamin D supplementation, an answer was obtained that contradicted the use of vitamin D .

3. Question about the number of days the patient sunbathes during the year, control question about wearing clothes that cover the whole body. The responses were considered inconsistent with obtaining information confirming sunbathing in a given patient simultaneously with the response about avoiding the sun by using clothing that covers the whole body.

4. Question about body weight and height from which BMI was calculated, as a control question was asked about overweight in postmenopausal age. Inconsistent responses were considered to be when the patients had BMI above the norm, with the patient's declaration that she was not overweight in postmenopausal age. Women who were pre-menopausal at the time of cancer diagnosis were excluded from the statistics for this study.

5. Question about the vitamin D test performed in the past during the second test cycle. The information that the patient had not been tested for vitamin D in the past was considered as inconsistent, which was contrary to the truth, because all the people participating in the study in the first cycle had a vitamin D test performed.

Depending on the question, the agreement was from 82 to 95%.
