# Supplementary material for: Effect of Selected Factors on the Serum 25(OH)D Concentration in Women Treated for Breast Cancer
Source: Nutrients. 2021 Feb 9;13(2):564. doi: 10.3390/nu13020564 (PMC7915136; doi:10.3390/nu13020564)
Supplement: Supplementary file 1 [file nutrients-13-00564-s001.zip › nutrients-1060971-supplementary materials/File S3 Personal data anonymization.docx]

Personal data anonymization

For the purpose of anonymization, the questionnaires did not use data such as names or addresses of participants. Each examined person was assigned an individual numerical code by which the persons participating in the research were identified. Only the person conducting the research was able to decode the obtained data. This was done for medical purposes only (i.e., to provide a Vitamin D result and relate the Vitamin D result to survey data).
