# Supplementary material for: Effect of Selected Factors on the Serum 25(OH)D Concentration in Women Treated for Breast Cancer
Source: Nutrients. 2021 Feb 9;13(2):564. doi: 10.3390/nu13020564 (PMC7915136; doi:10.3390/nu13020564)
Supplement: Supplementary file 1 [file nutrients-13-00564-s001.zip › nutrients-1060971-supplementary materials/Table S1 Anthropometric characteristics of the patients.docx]

Table S1. Anthropometric characteristics of the patients.

|  | **Age (ys)** | **Height (cm)** | **Body mass (kg)** | **BMI (kg/m^2^)** |
| --- | --- | --- | --- | --- |
| Group A (n = 62) | 64.5 ± 11.0 | 161.6 ± 5.9 | 70.2 ± 10.4 | 26.9 ± 4.1 |
| Group B (n = 32) | 63.0 ± 8.0 | 162.9 ± 6.7 | 74.0 ± 12.8 | 27.9 ± 4.4 |
| Control group (n = 93) | 62.1 ± 7.1 | 162.1 ± 6.3 | 68.9 ± 10.6 | 26.2 ± 3.7 |
| *p value* | *p* = 0.234 | *p* = 0.662 | *p* = 0.156 | *p* = 0.148 |

Notes: Group A – women treated for breast cancer tested first time in winter; Group B – women treated for breast cancer tested first time in summer; n—number of the patients; variables are presented as mean ± SD. *p* - ANOVA
