# Supplementary material for: Effect of Selected Factors on the Serum 25(OH)D Concentration in Women Treated for Breast Cancer
Source: Nutrients. 2021 Feb 9;13(2):564. doi: 10.3390/nu13020564 (PMC7915136; doi:10.3390/nu13020564)
Supplement: Supplementary file 1 [file nutrients-13-00564-s001.zip › nutrients-1060971-supplementary materials/Table S2 Characteristics of patients related to the reproductive system.docx]

Table S2. Characteristics of patients related to the reproductive system.

|  | **Age of First Menstruation**  **(ys)** | **Age of Menopause**  **(ys)** | **Number of Nulliparous**  **(%)** | **Age of First Childbirth**  **(ys)** | **Number of Patients with HRT (%)** |
| --- | --- | --- | --- | --- | --- |
| Group A  (*n* = 62) | 14.1 ± 1.6 | 50 (45; 51) | 8 (13) | 23.5 (21; 27) | 9 (14) |
| Group B  (*n* = 32) | 14 (13; 15) | 50 (49; 53) | 4 (13) | 23.5 (21; 25.5) | 4 (13) |
| Control group (*n* = 93) | 14.0 ± 1.6 | 50.5 ± 4.1 | 15 (16) | 24.4 ± 4.0 | 25 (26) |
| *p* | *p* = 0.652 | *p* = 0.657 | *p* = 0.85 | *p* = 0.699 | *p* = 0.04 |

Notes: Group A – women treated for breast cancer tested first time in winter; Group B – women treated for breast cancer tested first time in summer; HRT - hormone replacement therapy; *n*-sample size; for quantitative variables mean values ± standard deviation or median and quartiles are reported, for qualitative variables *n* and %; *p* - Friedman's rank test
