# Supplementary material for: Effect of Selected Factors on the Serum 25(OH)D Concentration in Women Treated for Breast Cancer
Source: Nutrients. 2021 Feb 9;13(2):564. doi: 10.3390/nu13020564 (PMC7915136; doi:10.3390/nu13020564)
Supplement: Supplementary file 1 [file nutrients-13-00564-s001.zip › nutrients-1060971-supplementary materials/Table S3 Charactersitics of the patients in relation to cancer disease.docx]

Table S3. Characteristics of patients related to the cancer disease.

|  | **Group A**  **(*n* = 62)** | **Group B**  **(*n* = 32)** | **Control group**  **(*n* = 93)** | ***p* – comparison group A vs B** |
| --- | --- | --- | --- | --- |
| Age at which breast cancer was diagnosed (ys) | 52.7 ± 11.8 | 53.4 ± 8.8 | - | *p* = 0.63 |
| Years from cancer detection to present study (ys) | 13.8 ± 11.5 | 11.3 ± 12.7 | - | *p* = 0.34 |
| Number of patients with complementary chemo- and/or hormone- therapy (percentage) | 25 (46.30) | 11 (35.5) | - | *p* = 0.57 |
| Number of patients with complementary radio-therapy (percentage) | 14 (22.58) | 10 (31.25) | - | *p* = 0.36 |
| Number of patients with cases of breast cancer in a close relatives (percentage) | 8 (12.9) | 4 (12.5) | 10 (10.7) | *p* = 0.93  **p* = 0.70 |
| Number of patients with cases other cancer in the family (percentage) | 18 (29.0) | 13 (40.62) | 29 (31.2) | *p* = 0.24  **p*= 0.85 |

Notes: Group A – women treated for breast cancer tested first time in winter; Group B – women treated for breast cancer tested first time in summer; *n*-sample size; variables are presented as mean values ± SD ; *combined group A + B vs control group. *p -*  t-test and Chi^2^ test, where appropriate.
