# Supplementary material for: Effect of Selected Factors on the Serum 25(OH)D Concentration in Women Treated for Breast Cancer
Source: Nutrients. 2021 Feb 9;13(2):564. doi: 10.3390/nu13020564 (PMC7915136; doi:10.3390/nu13020564)
Supplement: Supplementary file 1 [file nutrients-13-00564-s001.zip › nutrients-1060971-supplementary materials/Table S4 The frequency of consumption of foods rich in vitamin D.docx]

Table S4. The frequency of consumption of foods rich in vitamin D per week in the combined group A + B and control group, before entering the study.

|  | **Consumption per week** | **Number of patients from combined group A + B (n, %)** | **Number of patients from control group**  **(n, %)** | ***p*** |
| --- | --- | --- | --- | --- |
| Fish | < 1 | 27 (29) | 28 (30) | *p* = 0.84 |
|  | 1-2 | 37 (39) | 49 (53) | *p* = 0.07 |
|  | > 2 | 30 (32) | 16 (17) | *p* = 0.019 |
|  |  |  |  |  |
| Dairy | < 1 | 12 (13) | 9 (10) | *p* = 0.50 |
|  | 1-2 | 30 (32) | 42 (45) | *p* = 0.06 |
|  | > 2 | 52 (55) | 42 (45) | *p* = 0.14 |

Notes: number of patients in combined A and B group n= 94 (Group A – women treated for breast cancer tested first time in winter; Group B – women treated for breast cancer tested first time in summer), number of patients in control group n=93. Variables are presented as *n* – number of patients and % - percentage of indicated persons in the group; *p* – Chi^2^ test
