# Supplementary material for: Effect of Selected Factors on the Serum 25(OH)D Concentration in Women Treated for Breast Cancer
Source: Nutrients. 2021 Feb 9;13(2):564. doi: 10.3390/nu13020564 (PMC7915136; doi:10.3390/nu13020564)
Supplement: Supplementary file 1 [file nutrients-13-00564-s001.zip › nutrients-1060971-supplementary materials/Table S5 Number of patients consumed food rich in vitamin D.docx]

Table S5. Number of patients consumed food rich in vitamin D before first and second testing in combined A + B groups.

|  | < 1 per week  (*n*, %) | 1-2 x per week (*n*, %) | > twice per week (*n, %*) |
| --- | --- | --- | --- |
|  |  |  |  |
| Fish before first testing | 28 (30) | 37 (39) | 29 (31) |
| Fish before second testing | 35 (37) | 31 (33) | 28 (30) |
| Dairy before first testing | 8 (9) | 37 (39) | 49 (52) |
| Dairy before second testing | 19 (20) | 22 (23) | 53 (57) |

Notes: Group A – women treated for breast cancer tested first time in winter; Group B – women treated for breast cancer tested first time in summer; variables are presented as n – number of patients and % - percentage of indicated persons in the group.
